# Characterization key genes of Arabidopsis seedlings in response to β-caryophyllene, eugenol using combined transcriptome and WGCN analysis

**DOI:** 10.3389/fpls.2023.1295779

**Published:** 2024-01-04

**Authors:** Yuqi Guo, Chang Liu, Yaran Zhang, Shuting Zheng, Ping Cao, Xiaomin Wang, Zengyuan Tian

**Affiliations:** ^1^ School of Life Sciences, Zhengzhou University, Zhengzhou, Henan, China; ^2^ School of Agricultural Sciences, Zhengzhou University, Zhengzhou, Henan, China

**Keywords:** Arabidopsis, β-caryophyllene, eugenol, hormone, cell wall

## Abstract

Weeds present a significant challenge to high crop yield and quality. In our study, we investigated the phytotoxic activity of β-caryophyllene (BCP) and eugenol, which are natural allelopathic chemical compounds, on Arabidopsis seedlings. We found that these compounds inhibited the growth of *Arabidopsis thaliana* plants. When either BCP or eugenol was applied, it led to decrease in the content of cell wall components such as lignin, cellulose, hemicellulose, and pectin; and increase in the levels of endogenous hormones like ETH, ABA, SA, and JA in the seedlings. Through transcriptome profiling, we identified 7181 differentially expressed genes (DEGs) in the roots and shoots that were induced by BCP or eugenol. The genes involved in the synthesis of lignin, cellulose, hemicellulose, and pectin were down-regulated, whereas genes related to synthesis and signal transduction of ABA, ETH, SA, and JA were up-regulated. However, genes related to IAA synthesis and signal transduction were found to be down-regulated. Furthermore, we characterized 24 hub genes using Weighted Correlation Network Analysis (WGCNA). Among them, the identified 16 genes in response to BCP was primarily associated with hypoxia stress, while 8 genes induced by eugenol were linked to inhibition of cell division. Our results suggested that BCP and eugenol had ability to target multiple genes to inhibit growth and development of Arabidopsis plants. Therefore, they can serve as excellent candidates for natural biological herbicides.

## 1 Introduction

Weeds pose one of the most significant challenges to crop yields, resulting in significant losses in crop quality (Macias-Rubalcava and Garrido-Santos, 2022). Currently, synthetic herbicides are the primary tools used to control weeds. However, their low biodegradability and high persistence in soil have led to serious environmental risks, including contamination of surface and ground waters. Moreover, the number of herbicide-resistant weeds is continuously increasing. For instance, glyphosate-resistant and synthetic auxin herbicide (SAH) 2,4-D-resistant weed species have become increasingly common (Busi et al., 2018).

Using synthetic herbicides or breeding transgenic crops to control herbicides for weed management can lead to rapid evolution of herbicide-resistant weeds (Duke, 2015). Currently, there are 404 unique cases (species × site of action) of herbicide-resistant weeds globally (Heap, 2014). Thus, relying on synthetic herbicides or transgenic crops for weed management is unsustainable.

Most plants release cytotoxic chemicals into the environment, a process known as allelopathy to compete for resources with their neighbors. This mechanism helps plants to compete with neighboring organisms for limited nutrients (McCoy et al., 2022). Phenolic compounds, terpenoids, and benzoxazinoids are among the most important natural allelochemicals, which can be used as natural herbicides, are helpful in overcoming weed resistance to control weeds in sustainable agricultural systems. (Huang et al., 2010; Macias et al., 2019).

The sesquiterpene β-caryophyllene (BCP) is a major compound found in several plant species, including *Psidium guajava L*. (Ullah et al., 2021). It is used for remedy in several diseases (Sharma et al., 2016). Additionally, and caryophyllene oxide have been reported as potent anti-inflammatory agents, inducing apoptosis in these cells (Sain et al., 2014). In *Arabidopsis thaliana*, the miR156-SPL module regulates the formation of BCP during the flowering stage by modulating the expression of the sesquiterpene synthase gene *TPS21* (Yu et al., 2015).

Eugenol is a phenolic aromatic ingredient present in clove oil. It not only boosts the antioxidant capacity of cells (Stone, 2017), but only exhibits antimicrobial properties against a range of pathogenic bacteria (Da Camara et al., 2022; Fu et al., 2022). It can easily permeate the lipopolysaccharide layer of cell membranes, penetrate cytoplasmic membrane and cytoplasm of gram-negative bacteria, causing intracellular component leakage (da Silva et al., 2018). Additionally, eugenol has been shown to be highly phytotoxic to several plant species, including amaranth, ryegrass, quinoa, and barnyard grass (Evans et al., 2009), which inhibits the germination and early growth of wild oats, reduces plant photosynthetic efficiency and chlorophyll content, and induces the accumulation of excess ROS (Ahuja et al., 2015).

The application of allelochemicals to control weeds is a preferable method for resistant weeds in sustainable agricultural systems. The molecular mechanism that BCP or eugenol, as major allelochemicals, used to induce death of cells in plants remains unknown. In the present study, the effect of BCP or eugenol on the growth of *A. thaliana* seedlings was investigated. A combination of transcriptome and WGCN analysis was used to identify key genes, the results extend our knowledge related to the specific action modes of BCP/eugenol on plants.

## 2 Material and methods

### 2.1 Plant materials and growing conditions

The *A. thaliana* seeds were first sterilized by dipping them in 75% ethanol for 1 minute, followed by a 10-minute soak in a 5% NaClO solution. Afterwards, they were washed at least five to ten times with sterilized water. Next, the seeds were placed on Murashige and Skoog (MS) agar medium and stratified at 4° for three days. Subsequently, they were cultured for 15 days under photoperiod at 8 hours light/16 hours dark at 28°C. Following this, uniform seedlings were transplanted into pots containing sterilized perlite and vermiculite and irrigated with a ½ Hoagland solution once every two days for one week. Then the plants were irrigated with 1/2 Hoagland supplemented with 0 (control), 450, 900, and 1800μM of BCP or eugenol once every two days for 7 days. The shoot and root tissues were harvested separately, for analysis.

### 2.2 Measurement of chlorophyll, malondialdehyde, the relative electrolytic leakage, H_2_O_2_, O^2-^

Chlorophyll extraction was conducted using ethanol as a solvent (Adil et al., 2022). The Malondialdehyde (MDA) content of leaves and roots was estimated using the thiobarbituric (TBA) method, as described by (Xing et al., 2022). The relative electrolytic leakage was measured according to methods of Huang (Huang et al., 2021).

The accumulation of H_2_O_2_ was detected by DAB staining at room temperature (Wu et al., 2019). Fresh leaves of different treatment groups were soaked in buffer solution for 24 hours, and then immersed in 100 alcohol: oil (3:1) for 15 minutes. Finally, the leaves were photographed and recorded.

The content of superoxide anion was determined by hydroxylamine oxidation (Kozuleva et al., 2015). 0.2g sample was added to 2 mL 65mM phosphoric acid buffer(PH 7.8), and fully ground, and the supernatant was taken as the extraction solution of superoxide anion separation at 12000 rpm for 10 min. Take 1 mL of extract, add 0.75 ml of phosphoric acid buffer (pH 7.8) and 0.25 mL of 10 mM hydroxylamine hydrochloride. After 25 bath reaction for 30 min, continue to add 2 mL of 7 mM alpha-naphthylamine and 2 mL of 17 mM p-aminobenzenesulfonic acid, 30 OD value was measured at 530 nm after bath reaction for 30 min.

### 2.3 Determination content of pectin, lignin, cellulose and hemicellulose in shoots and roots

The isolation of cell walls from plants followed the method described by Hu et al. (Hu et al., 2018). First, fresh shoot or root samples were ground in a mortar with pre-cooled PBS (10 mM) buffer to obtain homogenization, which was then vacuum filtered. The resulting filtrate was washed and filtered four times with PBS buffer, followed by washing with acetone until the filtrate became colorless. The obtained filtrate was the cell wall fragments. These fragments were then extracted and dried at 65°C for future use.

Pectin was extracted by boiling 2 mg of cell wall extract in 1 ml of ultrapure water three times, each for 1 hour. After each boiling, the mixture was centrifuged at 4500 g for 3 minutes, and the supernatant was collected into one tube. The uronic acid content was measured using galacturonic acid as a reference, as described by Voragen et al. (Voragen et al., 2009). Specifically, 100 μl of the supernatant was mixed with 500 μl of 98% (v/v) H_2_SO_4_ containing 0.0125 M Na_2_B_4_O_7_ and boiled for 5 minutes. After cooling to room temperature, 10 μl of 0.15% (w/v) m-hydroxydiphenyl dissolved in 0.5% (w/v) NaOH was added, and the sample solution was incubated at room temperature for 15 minutes. The absorbance was measured at 520 nm using a spectrophotometer UV-1800PC.

To analyze the content of lignin, 0.1g of cell wall was added to 500 μl of 98% (v/v) H_2_SO_4_ and dissolved by adding 2 mL of acetyl bromo-glacial acetic acid (25%). The mixture was heated in a 70° water bath for 30 min, followed by addition of 0.9 mL of NaOH (2M) solution to terminate the reaction. After cooling to room temperature, 2 mL of glacial acetic acid and 0.1 mL of 5 M hydroxylamine hydrochloride were added. The volume was made constant to 7 mL with glacial acetic acid and the mixture was centrifuged at 4500 rpm for 5 min. Then, 200 μL of the supernatant was taken and the absorbance was measured at 280 nm using a spectrophotometer, as described by Hatfield et al. (Hatfield et al., 1999).

To measure the content of cellulose, 0.1g of the cell wall was incubated with 4 mL of cellulase (1 mg/L) for 37 hours under dark conditions. Then, 2 mL of 25% trichloroacetic acid was added and boiled for 10 minutes. The mixture was then centrifuged and the supernatant was collected. Cellulose content in the coarse cell wall was determined by the phenol-sulfuric acid method using glucose as a standard sugar. Briefly, 200 μL of the supernatant was mixed with 50 μL of 2% phenol and 3,500 μL of H_2_SO_4_ in a tube and boiled for 15 minutes. After cooling down to room temperature, the absorbance of the supernatant was measured at 490 nm (Hu et al., 2018).

The analysis of hemicellulose was carried out as follows. A 0.1g sample was mixed with 10 ml of PBS and boiled on a heater for 30 minutes. The resultant mixture was subjected to centrifugation and the supernatant was discarded. The pellets were rinsed thrice with distilled water, following which 10 ml of 4M KOH was added to the mixture and stirred at room temperature for 24 hours. The mixture was again centrifuged and the resulting supernatant was collected. The hemicellulose content in coarse cell wall was determined using the phenol-sulfuric acid method with glucose as the standard sugar (Sun et al., 2012).

### 2.4 Measurement of endogenous hormone in shoots and roots

The content of plant endogenous hormones (IAA, ETH, ABA, SA, JA) in *A. thaliana* seedlings was measured using ELISA kits. Endogenous hormones were extracted according to a modified method described by Chakraborty et al. (Chakraborty et al., 2013). Specifically, 0.1 g of shoots or roots tissues of each sample were ground with a mortar and pestle at liquid N_2_ in 0.9 mL of 50 mM PBS buffer. The extract was then centrifuged at 4000 rpm for 15 min and the supernatant was used to detect the concentration of hormones according to the instructions on the ELISA kit. A standard curve was obtained using standard hormones at different known concentrations (3, 6, 12, 24, and 48 pmol/L) and the concentrations of hormones produced were calculated by comparison with the standard curve.

### 2.5 RNA Extraction, library construction, and illumina sequencing

Total RNA was extracted using TRIzol reagent (Invitrogen, USA) according to the manufacturer’s instructions. Prior to library construction, total RNA was treated with RNase-free DNase I (New England Biolabs, USA) to remove any contamination of genomic DNA. After extraction, the RNA concentration was determined using a NanoDrop1000 spectrophotometer. The integrity of RNA was assessed by electrophoresis on a 1% agarose gel. The RNA samples with intact 28S and 18S ribosomal RNA bands were considered to be of good quality and suitable for downstream applications such as reverse transcription and PCR. The mRNA was enriched using oligo (dT) magnetic beads (Qiagen) and fragmented. First-strand cDNA was synthesized using a random hexamer primer, followed by the generation of second-strand cDNA using RNase H and DNA polymerase I. The resulting cDNA was purified, and end reparation and poly (A) addition were performed. Then, sequencing adapters were ligated to the cDNA, and the library was purified through agarose gel electrophoresis and enriched by PCR amplification to generate the final cDNA library. The cDNA libraries were sequenced on the Illumina HiSeq™ 2000 platform using paired-end technology at Yuanshen (Shanghai, China). The resulting clean RNA-seq reads were mapped to the *Arabidopsis* reference genome.

### 2.6 Data processing and differential expression

The normalized transcript abundance of the genes was calculated using the FPKM (Fragments Per Kilobase of transcript per Million mapped reads) method. Subsequently, differential expression analysis was conducted using edge R software. The false discovery rate (FDR) was used to determine the threshold of P value in multiple tests to control for false positives. In this study, a threshold of FDR ≤ 0.05 and log2|FC (ratio of BCP or Cary/control)| ≥ 1 were used to determine the significant differences in gene expression between the BCP or eugenol treated samples and the control.

Enrichment analysis of Gene Ontology (GO) terms was performed on significant DEGs using the AgriGO platform (http://geneontology.org/docs/go-enrichment-analysis/). Pathway analysis of Kyoto Encyclopedia of Genes and Genomes (KEGG) (http://david.abcc.ncifcrf.gov/, accessed on 15 April 2021) was used to elucidate significantly enriched pathways of the DEGs. In the present study, both GO terms and KEGG pathways with Q-values ≤ 0.05 were considered significantly enriched in DEGs. The heat map was plotted using the OmicShare tools (www.omicshare.com/tools).

### 2.7 Weighted gene co-expression network analysis

A matrix of normalized expression values of DEGs identified by p-value and FDR-based method was analyzed using the weighted gene correlation network analysis (WGCNA) package. Pearson correlations between pairs of DEGs were used to calculate a similarity matrix, and the results were transformed into an adjacency matrix using the following equation: aij = [0.5 ∗ (1 + cor(i, j)]^β^, where aij represents the connection strength between DEGs. The scale-free topology with R2 cutoff (0.8) was used to choose the soft-thresholding power beta of the co-expression network. Topological Overlap Matrix (TOM) was implemented to cluster DEGs, and a dynamic tree cut algorithm was used to construct gene co-expression modules. Two parameters, including containing at least 50 genes and a cut height higher than 40, were defined to select modules. Genes were clustered into 14 correlated modules.

To further study the gene modules associated with BCP or eugenol treatment, the correlation coefficients between module eigengenes and different samples were calculated. Modules with the highest correlation coefficients were selected as target gene modules, after BCP or eugenol treatment. GO and KEGG analysis is performed for genes in the selected target module. Gene significance (GS) and module correlation degree (MM) of each gene was calculated by the R package and genes with high connectivity tended to be hub genes which may have important functions. Network visualization for target module was performed using the Cytoscape software version 3.9. The gene co-expression network is a scale-free weighted gene network with multiple nodes connected to different nodes via edges. Each node represents a gene, which is connected to a different number of genes. The gene which is connected to a greater number of genes is denoted with a bigger size and is more important for its interaction with a large number of genes.

### 2.8 qRT-PCR confirmation of the RNA-seq

The RNA-seq data’s reliability was validated by means of qRT-PCR. RNA extraction and purification were undertaken, followed by cDNA synthesis using the RevertAid™ Kit (Fermentas). 16 genes were selected randomly for qRT-PCR analysis. To normalize the cDNA levels in each reaction, *A. thaliana* Actin gene was employed as endogenous control for relative expression calibration. **Supplementary Table 1** contains the primer sequences utilized for the genes selected. Maxima SYBR Green Master Mix (Thermo Scientific) was used for qRT-PCR, which was conducted using the Real-time Quantitative PCR System (iQ5, Bio-Rad, USA), with three repetitions performed. The 2^−ΔΔCT^ method was utilized to analyze the relative expression data.

### 2.9 Statistical analysis

All experiments were performed in triplicate, and the results were presented as mean ± standard error of the mean (SEM). Statistical analysis of experimental data was performed using GraphPad Prism 8.0 software and SPSS 25.0 software. Data were subjected to analysis of variance using one-way ANOVA, and statistical differences between control and treated groups were determined. P-values less than 0.05 were considered statistically significant.

## 3 Results

### 3.1 Effect of both BCP and eugenol on plant physiological parameters

#### 3.1.1 Both BCP and eugenol inhibited growth of *A. thaliana* plants and damaged their integrity of cells

Our results revealed that BCP or eugenol caused wilting symptoms and obviously inhibited the growth of the seedlings compared to the control group (**Supplementary Figures 1A, B**). The number of branches and leaves, fresh weight, root length, and plant height were significantly reduced after treatment with BCP or eugenol, except for the lower concentration of BCP or eugenol (100 μM) (**Supplementary Figures 1C-F**).

Treatment with BCP or eugenol (450, 900, 1800 μM) reduced the content of chlorophyll (**Supplementary Figure 2C**) in leaves and significantly increased the levels of REC (**Supplementary Figure 2A**), MDA (**Supplementary Figure 2B**), O_2_^-^ (**Supplementary Figure 2D**), and H_2_O_2_ (**Supplementary Figure 2E**) in both shoots and roots of *A. thaliana* seedlings compared to the control, except for treatment with concentration at 100 μM BCP or eugenol. These findings indicated that BCP or eugenol caused severe damage to the cell membrane integrity, ultimately affecting the normal growth of seedlings.

#### 3.1.2 BCP and eugenol damaged on components of cell wall in *A. thaliana* seedlings

**Figure 1:**
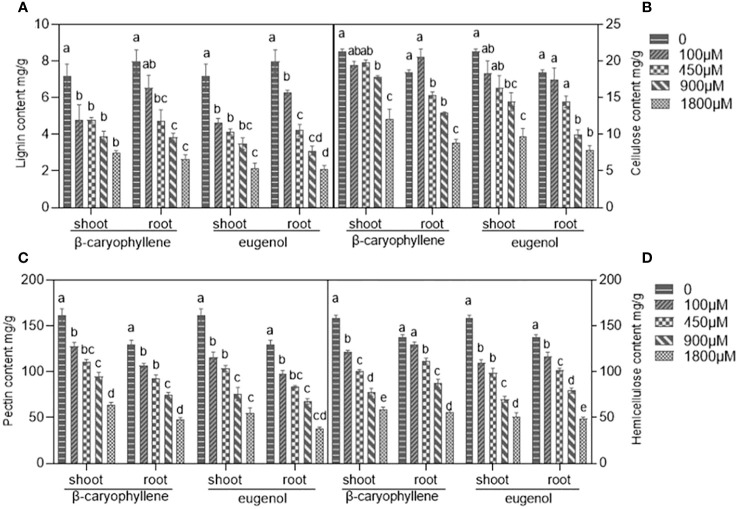
The effects of BCP or eugenol on lignin **(A)**, cellulose **(B)**, pectin **(C)** and hemicellulose **(D)** content in *Arabidopsis thaliana* seedlings. Different lower letters indicate the significant differences of multiple comparisons (P<0.05) using the least significant difference (LSD) method.

After treatment with BCP or eugenol, the content of lignin, pectin, cellulose and hemicellulose, which are the main components of plant cell wall, were decreased in roots and shoots of *A. thaliana* seedlings compared to control groups (**Figure 1**). The results suggested that both BCP and eugenol affected the injured the structure and stability of the cell wall, leading to damage to the plant cell.

#### 3.1.3 Effects of BCP or eugenol on hormone content of *A. thaliana* seedlings

**Figure 2:**
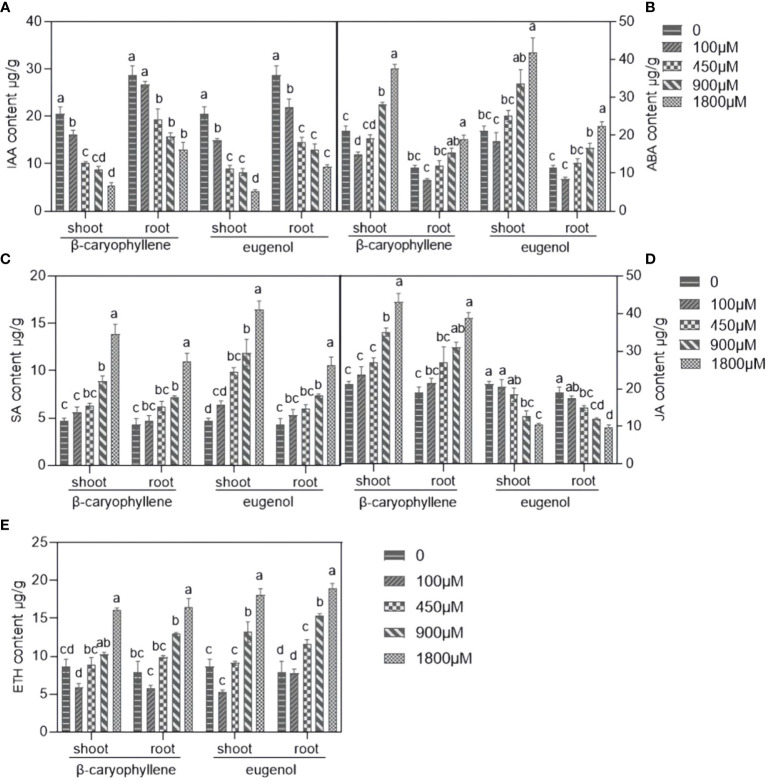
The effects of BCP or eugenol on IAA **(A)**, ABA **(B)**, SA **(C)**, JA **(D)** and ETH **(E)** content in *Arabidopsis thaliana* seedlings. Different lower letters indicate the significant differences of multiple comparisons (P<0.05) using the least significant difference (LSD) method.

As shown in **Figure 2**, the content of phytohormones, including ABA, SA, and ETH in the shoots and roots of seedlings were significantly increased after treatment with BCP or eugenol compared to the control group, except for the IAA, which decreased. The JA content in the shoots and roots of the seedlings increased significantly in response to BCP but decreased in response to eugenol. These results indicated that BCP or eugenol severely disrupted hormone balance of *A. thaliana* seedlings.

### 3.2 Differential gene expression of *A. thaliana* in response to BCP or eugenol

We conducted transcriptome analysis of 18 samples of *A. thaliana* in roots and shoots under treatment with 0 and 900 μM BCP or eugenol using Illumina paired-end sequencing, resulting in a total of 56.93 GB of clean data. Each sample provided 6.48 GB of data with a Q30 base percentage of 93.43% or higher. The alignment efficiency of each sample’s Clean Reads with the defined reference genome ranged from 86.40% to 93.73%.

**Figure 3:**
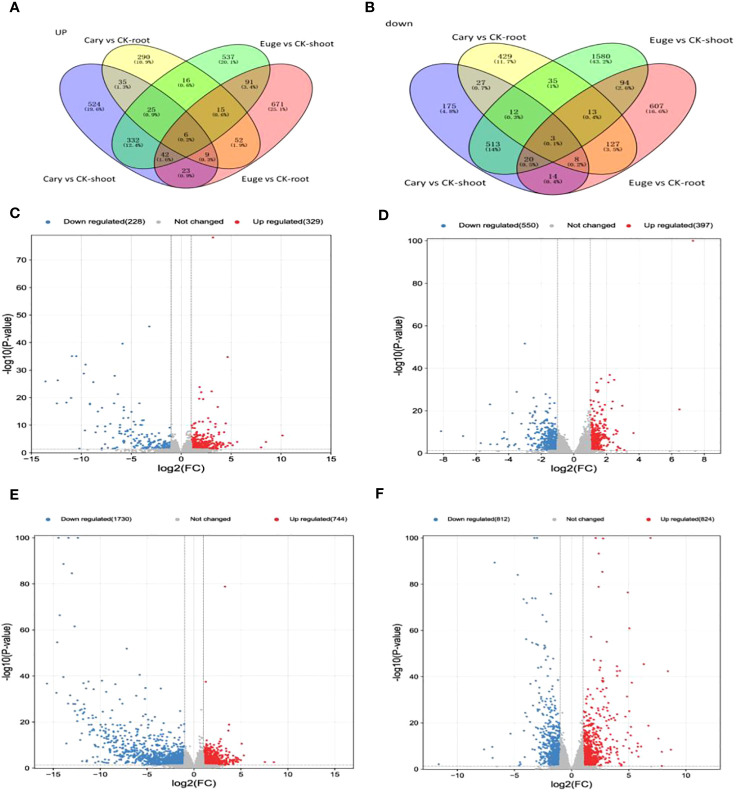
Venn diagram for DEG upregulated **(A)** and downregulated **(B)** after BCP treatment or eugenol treatment; Volcanic plot of DEGs in shoot **(C)** and root **(D)** after BCP treatment. Volcanic plot of RNA-Seq data using log_2_ fold change in shoot **(E)** and root **(F)** by eugenol treatment. Red and blue dots denote up- and down-regulated genes, respectively, Gray dots denote genes with no significant expression.

There were 2620 differentially expressed genes (DEGs) in response to BCP in the *A. thaliana* seedlings, among which, 921 and 374 genes were up-regulated, 722 and 603 genes were down-regulated in shoots and roots respectively. There were 4561 DEGs to eugenol, among which, 910 and 755 genes were up-regulated, but 2140 and 756 gene down-regulated in shoots and roots, respectively (**Figure 3**).

### 3.3 Gene ontology classification and KEGG pathway enrichment of DEGs

The identified DEGs were annotated into the three main GO functional categories, including cellular component (CC), molecular function (MF), and biological process (BP). According to the GO annotations, unigenes were classified into different functional categories.

**Figure 4:**
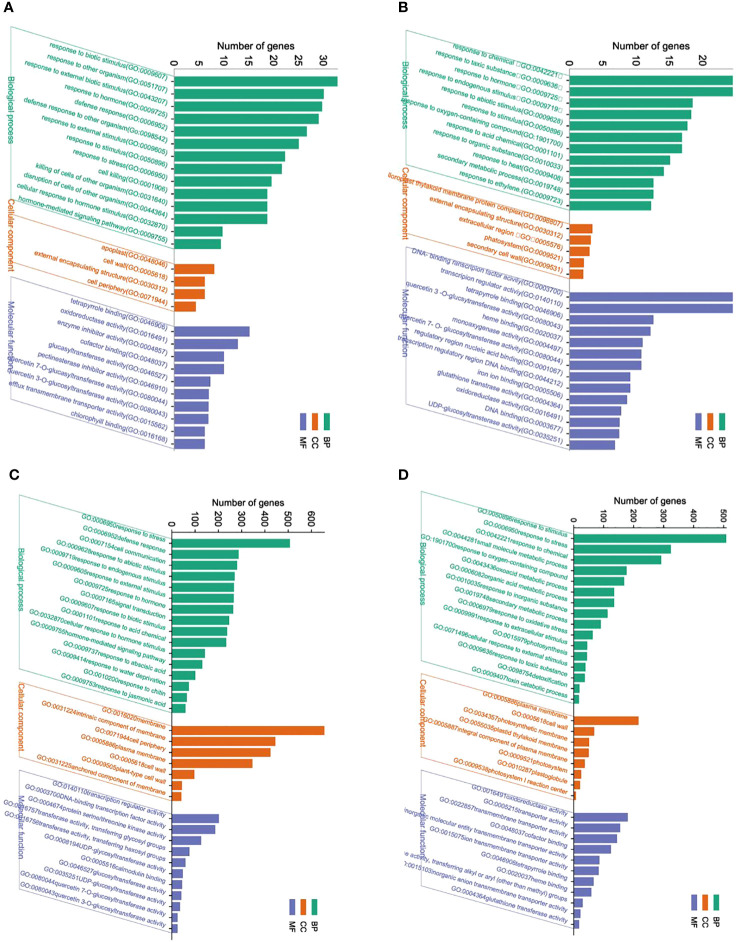
GO functional annotation and classification of DEG of shoot **(A)** and roots **(B)** in *A. thaliana* seedlings after BCP or GO functional annotation and classification of DEG of shoot **(C)** and roots **(D)** in *A. thaliana* seedlings after with eugenol treatment.

The GO enrichment analysis showed significant differences in the functional enrichment of genes in response to BCP (**Figures 4A-D**). DEGs in shoots treated with BCP were categorized into five major sub-categories, including “response to biotic stimulus (GO:0009607),” “response to other organisms (GO:0051707),” “response to external biotic stimulus (GO:0043207), response to hormone (GO:0009725) “ and “defense response (GO:0006952), in the “biological process” category.” Moreover, cellular component category contained terms such as “apoplast (GO:0048046),” “cell wall (GO:0005618),” “external encapsulating structure (GO:0030312),” and “cell periphery (GO:0071944).” In the “molecular function” category, the most abundant sub-categories were “tetrapyrrole binding (GO:0046906),” “oxidoreductase activity (GO:0016491),” and “enzyme inhibitor activity (GO:0004857)” (**Figure 4A**). Regarding the GO annotation of DEGs in roots, the “biological category” showed that the most DEGs were associated with five subterms: “response to chemical (GO:0042221) “, “response to toxic substance (GO:0009636) “,”response to hormones (GO:0009725),” and “response to endogenous stimulus(GO:0009719), “response to abiotic stimulus GO:0009628”.”The “cell component” category contained three main sub-categories, which were “chloroplast thylakoid membrane protein complex (GO:0098807),” “external encapsulating structure (GO:0030312),” and “extracellular region (GO:0005576).” For the “molecular function” category, the most DEGs were associated with “DNA-binding transcription factor activity (GO:0003700),” “transcription regulator activity (GO:0140110),” “tetrapyrrole binding (GO:0046906),” and “quercetin 3-O-glucosyltransferase activity (GO:0080043)” (**Figure 4B**).

Upon treatment with eugenol, DEGs in shoots were associated with three major sub-categories in the biological process category: “response to stress (GO:0006950)”, “defense response (GO:0006952)”, and “cell communication (GO:0007154)”. The most prevalent terms in the cell component category were “membrane (GO:0016020)”, “intrinsic component of membrane (GO:0031224)”, and “cell periphery (GO:0071944)”. In the molecular function category, the three main terms were “transcription regulator activity (GO:0140110)”, “DNA-binding transcription factor activity (GO:0003700)”, and “protein serine/threonine kinase activity (GO:0004674)” (as shown in **Figure 4C**). For roots, DEGs were predominantly assigned into the biological process category, where the three most abundant terms were “response to stimulus (GO:0050896)”, “response to stress (GO:0006950)”, and “response to chemical (GO:0042221)”. The cell component category primarily involved three sub-categories: “plasma membrane (GO:0005886)”, “cell wall (GO:0005618)”, and “photosynthetic membrane (GO:0034357)”. The most prevalent sub-categories in the molecular function category were “oxidoreductase activity (GO:0016491)”, “transporter activity (GO:0005215)”, and “transmembrane transporter activity (GO:0022857)” (as illustrated in **Figure 4D**).

**Figure 5:**
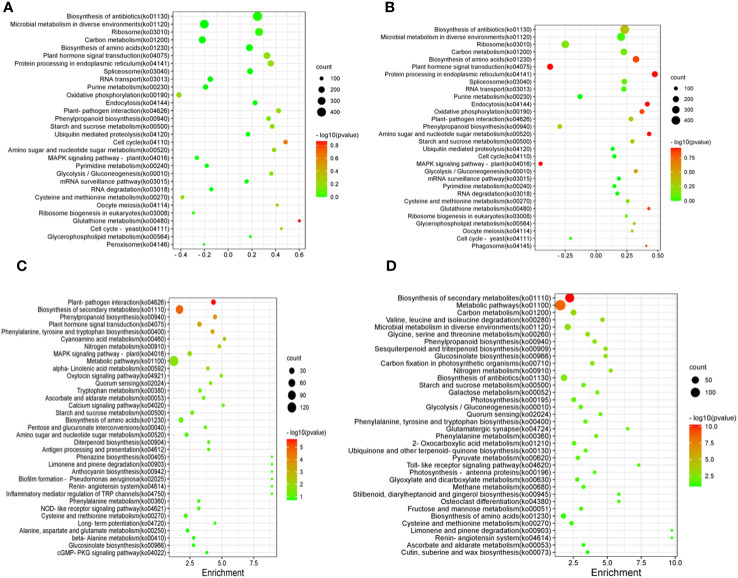
Differentially expressed Gene KEGG pathway enrichment of shoot in *A. thaliana* seedlings after BCP **(A)** or eugenol **(C)** treatment and differentially expressed Gene KEGG pathway enrichment of root in *A. thaliana* seedlings after BCP **(B)** or eugenol **(D)** treatment.

The results of KEGG enrichment analysis of shoots and roots after BCP treatment were presented in **Figure 5A**. The DEGs were distributed across 30 pathways in the shoots. The significant pathways included glutathione metabolism (ko00480), cell cycle (ko04110), cell cycle-yeast (ko04111), plant-pathogen interaction (ko04626), plant hormone signal transduction (ko04075), oxidative phosphorylation (ko00190),biosynthesis of antibiotics (ko01130), microbial metabolism in diverse environments (ko01120), and ribosome(ko03010).Similarly, the detected DEGs in roots were mainly enriched in biosynthesis of amino acids (ko01230), plant hormone signal transduction (ko04075), biosynthesis of antibiotics (ko01130), microbial metabolism in diverse environments (ko01120), Ribosome (ko03010) and plant hormone signal transduction (ko04075) (**Figure 5B**).

After treatment with eugenol, the KEGG enrichment analysis revealed that DEGs in shoots of *A. thaliana* seedlings were enriched in 35 pathways. The major pathways included plant-pathogen interaction (ko04626), biosynthesis of secondary metabolites (ko01110), phenylpropanoid biosynthesis (ko00940), plant hormone signal transduction (ko04075), phenylalanine, tyrosine and tryptophan biosynthesis (ko00400), and cyanoamino acid metablism (ko00460) (**Figure 5C**). DEGs in roots were enriched mainly in biosynthesis of secondary metabolites (ko01110), metabolic pathways (ko01100), carbon metabolism (ko01200), valine, leucine and isoleucine degradation (**Figure 5D**).

Both BCP and eugenol inhibited weed growth by inhibiting the biosynthesis of cell wall, while eugenol also damaged production of other secondary metabolites. They had diverse effects on the *A. thaliana* seedlings, affecting multiple pathways and processes.

### 3.4 DEGs involved in phenylpropanoids-related pathway and their expression pattern

**Figure 6:**
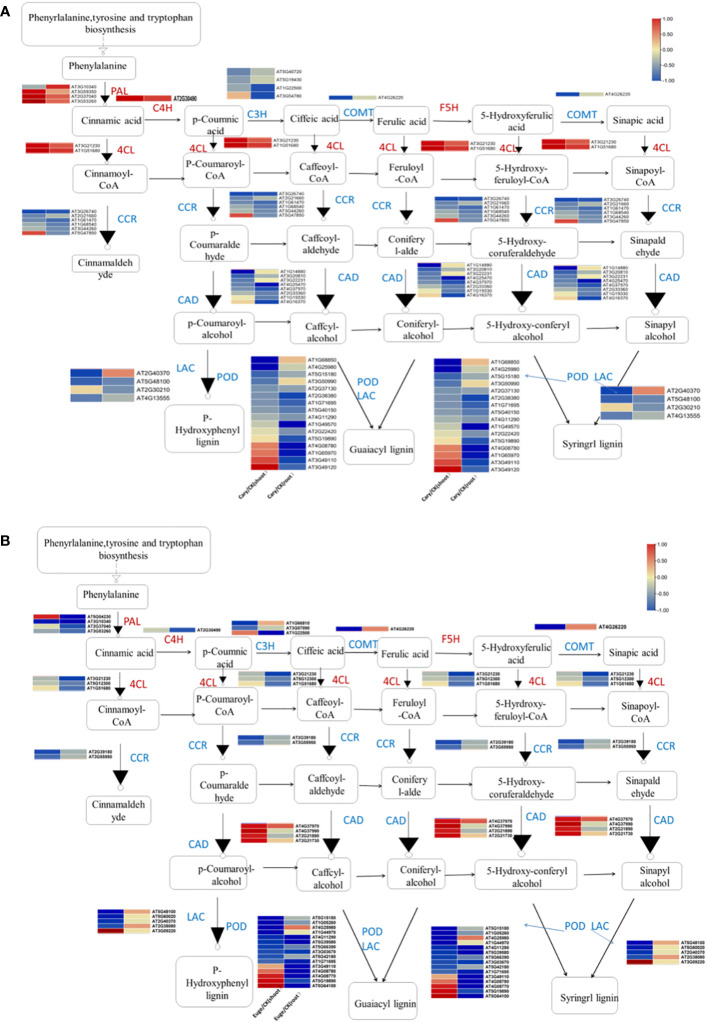
Analysis of genes expression related to phenylpropionic acid biosynthesis in *A. thaliana* seedlings by BCP **(A)** or eugenol **(B)** treatment.

Phenylpropanoids are various natural phenolic compounds in plants, which serve as vital structural components of cell walls and function as phytoalexins in protecting plants from herbivores and pathogens. Phenylpropanoid biosynthesis produces precursors for a wide range of phenolic compounds, including ferulic acid, p-coumaric acid, and caffeic acid (Glagoleva et al., 2022). A total of 47 and 39 DEGs, which associated with phenylpropanoid biosynthesis were identified in response to BCP or eugenol, respectively. These DEGs included genes involved in various process of the phenylpropanoid biosynthesis pathway such as phenylalanine ammonia lyase (PAL), caffeic acid 3-O-methyltransferase (COMT), trans cinnamate 4-monooxygenase (C4H), cinnamol dehydrogenase (CADH), 4-coumaric acid CoA ligase (4CL), laccase (LAC), coumaryl-3-hydroxylase (C3H), peroxidase (POD), cinnamyl-CoA reductase (CCR), and glucosyl transferase (UGT) (De Vries et al., 2021). Most of these DEGs, including *C3H*, *CCR*, *COMT*, and *LAC* genes, were down-regulated in both the shoots and roots after treatment with BCP or eugenol. However, the *PAL*, *C4H*, and *4CL* genes were significantly up-regulated in response to BCP, but down-regulated in response to eugenol in both shoots and roots. Additionally, the cinnamyl alcoholdehydrogenase (*CAD*) gene was down-regulated under BCP treatment, while it was up-regulated in both the shoots and roots with eugenol treatment (**Figures 6A, B**).

**Figure 7:**
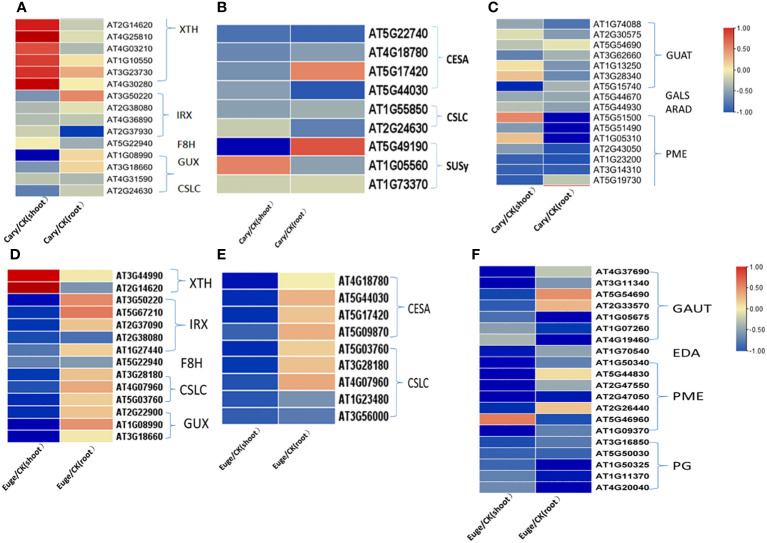
Analysis of gene expression related to the synthesis and metabolism of cell wall components hemicellulose **(A)**, cellulose **(B)** and pectin **(C)** of *A. thaliana*, seedlings by BCP treatment; Eugenol treatment for the expression of hemicellulose **(D)**, cellulose **(E)**, pectin **(F)** cell wall components hemicellulose **(D)**, cellulose **(E)**, pectin **(F)** related gene expression of *A. thaliana* seedlings.

The DEGs involved in the metabolism and biosynthesis of cell wall components were further analyzed. BCP or eugenol induced in the down-regulated expression of most DEGs in the biosynthesis of hemicellulose, pectin, and cellulose in both shoots and roots. Xylose and xyloglucan are the primary components of hemicellulose in dicotyledons. In *A. thaliana*, genes of the glycosyltransferase 43, 47 (GT43, GT47) family member irregular xylem(IRX), fra8 homolog(F8H), glucuronic acid replacement of xylan (GUX) are associated with xylan biosynthesis. Xyloglucan is mainly synthesized by cellulose synthetase similar to cellulose synthase like-C (CSLC) (Pitaksaringkarn et al., 2014; Sewelam et al., 2021), but xyloglucanendo-transglucosylase/hydrolase (XTH) catalyzes xyloglucan polymers to hydrolyze. BCP or eugenol lead to down-regulated expression of genes including *IRX*, *F8H*, *GUX*, and *CSLC* for hemicellulose synthesis, while the *XTH* family genes up-regulated in the shoots (**Figures 7A, D**).

The biosynthesis of cellulose is catalyzed by cellulose synthase (CesA), cellulose synthase-like (CSL), and sucrose synthase (SUSy). In response to BCP or eugenol, some genes of *CesA*, and *CSL* and *SUSy*, were significantly down-regulated in the shoots and roots of seedlings (**Figures 7B, E**). Pectin in the cell wall is composed of homogalacturonan (HG), rhamnogalacturonan-I (RGI), and rhamnogalacturonan-II (RGII). Pectin synthesis involves the participation and regulation of various enzymes, including UDP-GlcA synthesis enzymes, galacturonosyltransferase (GAUT) protein family, and pectin methylesterase (PME), β-1,4galactosyltransferase (GALS), arabinosyltransferase (ARAD) and polygalacturonase (PG). The coordinated action of these enzymes enables the formation of different types of pectin in plant cell walls. Most genes of *GAUT*, *PME* and *PG* in shoots and roots were significantly down-regulated after exposure to BCP or eugenol (**Figures 7C, F**).

### 3.5 DEGs related to plant hormone signaling pathway

**Figure 8:**
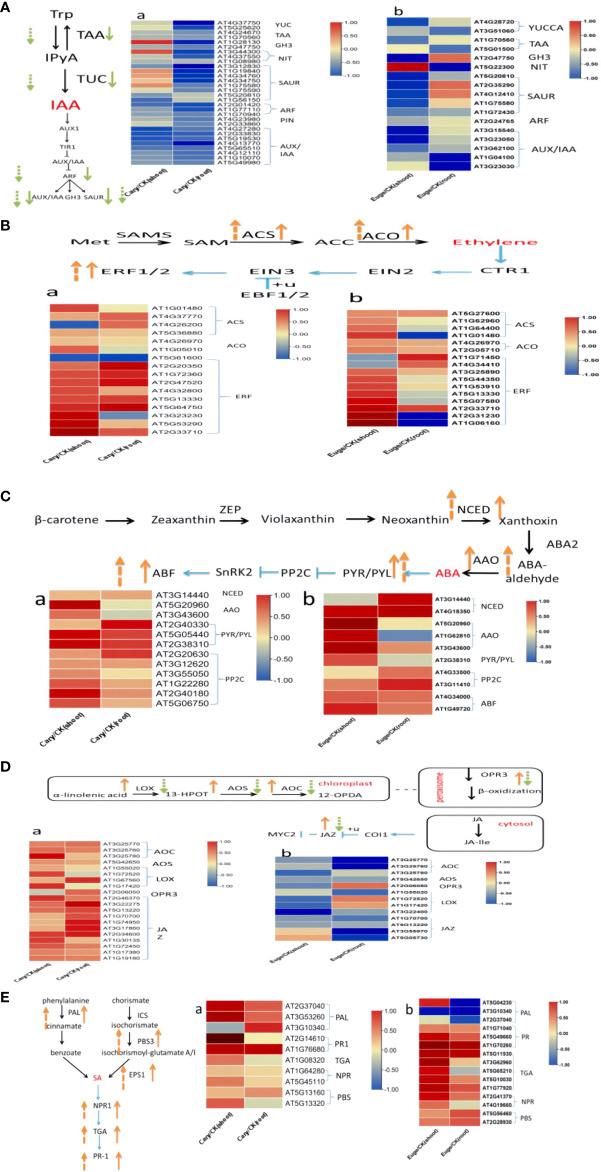
BCP **(A)** and eugenol **(B)** treatment for the analysis of DEGs in *A*. *thaliana* endogenous hormone auxin **(A)**, ethylene **(B)**, abscisic acid **(C)**, jasmonic acid **(D)** and salicylic acid **(E)** synthesis and signal transduction, respectively. The orange arrow in the figure indicates gene up-regulation, and the green arrow indicates down-regulation; the solid arrow represents BCP treatment, and the dotted arrow represents eugenol treatment.

Previous research indicates that tryptophan, carotenoid, cysteine, and methionine, α-linolenic acid, and phenylalanine serve as the biosynthetic precursors for auxin, abscisic acid, ethylene, jasmonic acid, and salicylic acid, respectively (Zhao, 2018). In this study, our results characterized DEGs, which are enzymes involved in precursors biosynthesis or signaling transduction pathways including IAA, ABA, SA, JA, and BR in Arabdopsis seedlings in the response to BCP or eugenol (**Figures 8A-E**). The DEGs involved in IAA pathways in plants comprise flavin monooxygenase (YUCCA), tryptophan transaminase (TAA), and nitrilase (NIT) (Yu et al., 2022), which were down-regulated. Additionally, genes such as *Auxin response factors (ARF)*, *Auxin/indole acetic acid (AUX/IAA)*, *Gretchen Hagen3 (GH3)*, and *Small auxin up RNA (SAUR)*, which participate in IAA signal transduction at the earlier stages (Pellizzaro et al., 2020; de Figueiredo et al., 2022), were also down-regulated in the roots and shoots after treatment with BCP or eugenol, especially in roots (**Figure 8A**).

The genes *ACC synthase (ACS)* and *ACC oxidase (ACO)* encode enzymes which catalyze the synthesis of ethylene (Li et al., 2020a), these genes were significantly up-regulated in response to BCP or eugenol. Additionally, transcription factors (ERFs) that play a role in the transmission of the ethylene signal were also up-regulated (**Figure 8B**).

The genes of 9-cis-epoxy dioxygenase (NCED) and *acetic aldehyde oxidase (AAO)*, which are related to ABA synthesis (Fan et al., 2009), were up-regulated in both shoots and roots after treatment with BCP or eugenol. Similarly, *ABA receptor gene pyrabactin resistance 1-like (PYL)* and *protein phosphatase 2C (PP2C)* gene were up-regulated as well (**Figure 8C**). The key genes, in the process of jasmonic acid synthesis including lipoxygenase (LOX), allene oxide cyclase (AOC), 12-oxo plant dienoic acid reductase (OPR3), and acyl coenzyme A oxidase (ACX) (Fidyt et al., 2016), were all up-regulated after treatment with BCP. However, the expression of *LOX, AOC, OPR3*, and *allene oxide synthase (AOS)* genes was down-regulated with eugenol. Jasmonate ZIMdomain (JAZ), a protein localized in the cell nucleus that plays a role in jasmonic acid signal transduction, was up-regulated after treatment with BCP but down-regulated with eugenol (**Figure 8D**). The genes of *phenylalanine ammonia lyase (PAL)* and *aminotransferase (PBS3)*, which are related to salicylic acid synthesis (Vlot et al., 2009), were significantly up-regulated after treatment with BCP or eugenol. Key genes, involved in disease resistance including *pathogenesis-related1 (PR1), TGACG-binding factor (TGA)*, and *nonexpressor of PR genes (NPR)* in the SA signal transduction pathway, were also up-regulated (**Figure 8E**).

### 3.6 Weighted gene co-expression network construction and module identification

**Figure 9:**
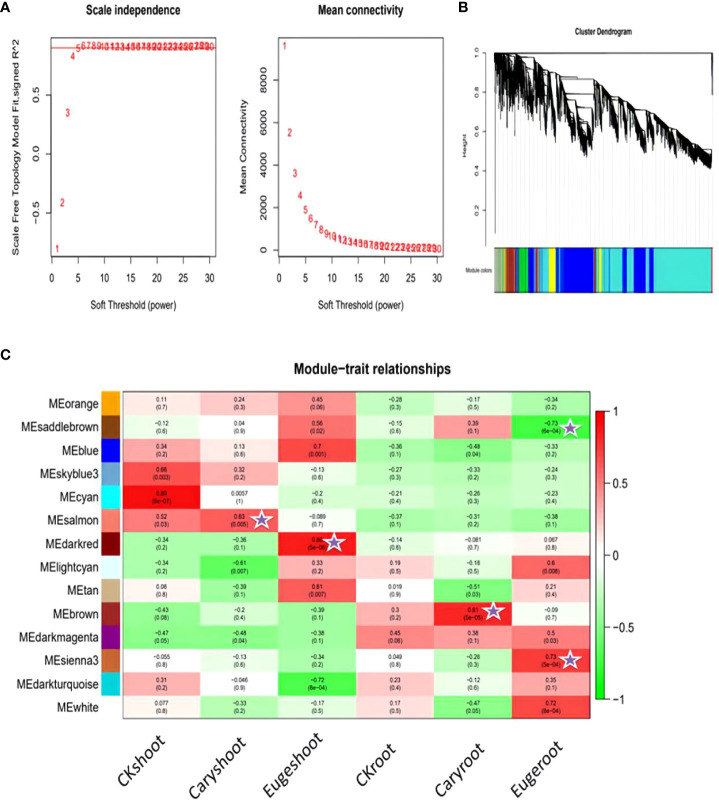
Association analysis of gene co-expression network modules with physiological and biochemical traits: **(A)** Soft threshold determination **(B)** Module detection by gene cluster dendrograms **(C)** Module-trait associations revealed by Pearson correlation coefficient.

To identify different co-expressed modules under treatment with BCP or eugenol in *A. thaliana*, we conducted WGCNA analysis to construct the gene co-expression network. We performed gene expression clustering tree and hierarchical clustering tree analyses on all 18 samples to calculate correlation coefficients for each sample’s expression level. The soft threshold β=5 was determined when the fitting curve was close to 0.8 for the first time (**Figure 9A**). The modules with similar expression were merged by the dynamic cutting tree method.

As a result, DEGs of different groups were assigned into 14 modules (**Figure 9B**), with the largest module containing 2693 genes and the smallest module containing 59 genes. We analyzed the correlation between modules and samples, and found that the salmon and brown modules were highly correlated with shoot and root of the seedlings after treatment with BCP, respectively. Similarly, the darked and sienna3 modules were highly correlated with shoot and root of the seedlings after eugenol, respectively (**Figure 9C**).

### 3.7 Functional annotation of candidate hub genes

**Figure 10:**
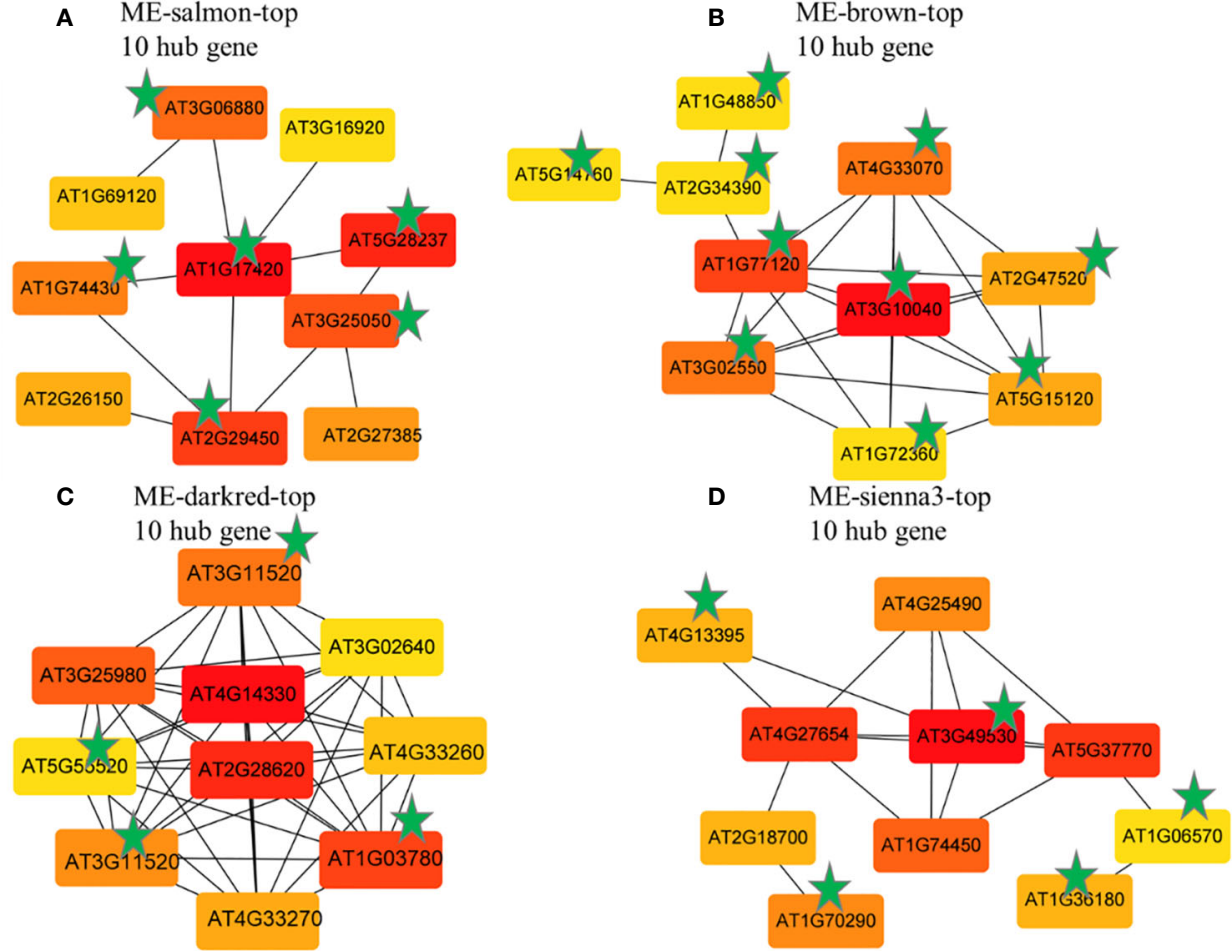
Top hub gene co-expression network of the MEsalmon **(A)**, MEbrown **(B)**, MEdarkred **(C)** and sienna3 **(D)** according to the degree value Note: The asterisk indicates the first 10 Hub genes shared by the degree algorithms and GS value.

The hub genes in each module with the most significant differential expression pattern across control and treatment were analyzed using Cytoscape. The genes in the target module were sorted using the degree algorithm and GS value, and the top 10 hub genes were selected to construct the core gene network diagram. There were 5, 9, 9, and 10 genes in the Salmon, Brown, Darked, and Sienna3 modules, respectively (**Figures 10A-D**). A total of 24 genes (**Supplementary Table S2**) with high connectivity in the four target gene modules were identified as candidate hub genes. The functions of these hub genes were annotated by using the NCBI and TAIR databases.

Among the identified 16 genes in response to BCP, 7 up-regulated genes which encode HRA1 (hypoxia response attenuator1), ADH1 (alcohol dehydrogenase), DUF1637 (ADO, 2-aminoethanethiol dioxygenase), ERF71 (ethylene-responsive transcription factors), ERF73, PDC1 (pyruvate decarboxylase1), and LBD41 (LOB domain-containing protein) respectively, were related to hypoxia. Other up-regulated genes, including *aspartate oxidase (AO)*, *xyloglucan endotransglucosylase/hydrolase 3(XTH3)*, and *lipoxygenase 3 (LOX3)*, were required for the ROS burst, abscission of organs, and cell separation, respectively. The genes down-regulated contained *AT3G06880, GSTU5 (glutathione S-transferase class tau 5), AT5G28237 (MYB95)*, and *NIP2.1*, all of which played a pivotal role in plant survival. These results suggest that BCP can lead to plant hypoxia stress, which caused excessive ROS, thus brought about lethal effects on plants.

Among the 8 hub genes induced by eugenol, 4 genes were associated with cell division, including *CDC20.1 (Cell Division Cycle 20), Cyclin B1-3(CYCB1), TPX2 (Targeting protein for Xklp2)*, and *AT5G55520* (unknown function), which were down-regulated. The other 4 genes, including *TPS8 (trehalose-6-P synthesis), PDS1(4-hydroxyphenylpyruvate dioxygenase), NAC062 (NAC domain-containing protein 62)* and *ACCase (Acetyl- coenzyme A carboxylase)*, which involved in response to external or internal environmental stress, were up-regulated. These results suggested that eugenol inhibited of cell division to lead to deleterious effects, caused the death of plants.

### 3.8 Real-time RT-PCR validation of differentially expressed genes

Real-time RT-PCR (qRT-PCR) was performed on 16 DEGs in response to BCP or eugenol. The results from sequencing and qRT-PCR were statistically analyzed, and a high correlation was observed with Pearson correlation coefficient R≈0.97 and R > 0.90 (**Supplementary Figure 3**). These results indicate that the transcriptome analysis accurately reflected the differential expression of *A. thaliana* genes under BCP or eugenol treatment, confirmed the reliability of the transcriptome data.

## 4 Discussion

### 4.1 The application of BCP and eugenol disrupted the endogenous hormone balance of plants, resulting in dysregulation of gene expression and regulation during growth and development

As natural compounds, both BCP and eugenol, were found to damage plant cell wall components, leading to a decrease in the plant’s ability to maintain cell shape and resist mechanical stress. These findings were consistent with transcriptome and WGCNA analyses, which demonstrated a significant down-regulation of genes involved in cell wall formation pathways, including lignin, cellulose, hemicellulose, and pectin biosynthesis (Hu et al., 2022). In addition, the expression of *XTH*, responsible for cell wall degradation, was found to be up-regulated. The down-regulation of genes related to cell wall component biosynthesis led to a reduced accumulation of lignin, pectin, cellulose, and hemicellulose, ultimately resulting in a decreased growth rate, developmental abnormalities, or even plant death when treatment with BCP or eugenol.

The growth and development of plant species can be disrupted by natural compounds derived from other plants, which affect their hormone balance. Lee et al. (2015) and Yan et al. (2020) conducted research that revealed how phenolic substances from locust seed extract inhibited the gibberellin pathway and promoted the accumulation of ABA, JA, and SA in plants. Similarly, Zhang et al. (2021) demonstrated that leaf aqueous extract reduced the IAA and GA content in grass seedlings, while increasing the ABA content. Plant stress responses are regulated by hormones such as IAA, ABA, JA, and ethylene (Yang et al., 2008; Li et al., 2020b). The biosynthesis and transport of these hormones are vital for plants to withstand abiotic and biotic stresses (Yang et al., 2008; Chen et al., 2020). However, an excess of hormones can have negative effects. For instance, excessive ABA can result in stomatal closure, reduced photosynthetic rate, and the generation of ROS, ultimately hindering plant growth and triggering senescence (Fan et al., 2009). Similarly, excessive SA can impede electron transport, induce stomatal closure, and inhibit photosynthesis (Ye et al., 2013). Although JA regulates various plant responses, including root growth inhibition, anthocyanin accumulation, and leaf senescence (Mori and Schroeder, 2004), an excessive accumulation of JA can cause stomatal closure, further reducing photosynthesis (Ye et al., 2013). In our study, the application of BCP or eugenol increased ROS levels and significantly altered endogenous hormone levels. The content of ABA, ethylene, JA, and SA increased significantly, while IAA decreased substantially. This disruption of the plant’s endogenous hormone balance inhibited plant growth.

Accordingly, the expression of key enzyme genes involved in the biosynthesis and transduction of hormones such as ethylene, ABA, JA, SA, and IAA in multiple metabolic pathways, was induced in response to BCP and eugenol treatments. This suggested that the application of BCP and eugenol externally disrupted the balance of plant endogenous hormones, leading to disorders in gene expression and regulation during growth and development.

### 4.2 BCP caused excessive accumulation of ethylene and low oxygen conditions, disrupting metabolic pathways and reducing the plant’s ability to withstand adverse environments while reducing the plant’s ability to detoxify

Characterization of the hub genes provided insight into the molecular mechanism of allelopathy between plants and adjacent organisms. Based on the 16 hub genes that responded to BCP, it can be inferred that BCP leads to excessive ethylene accumulation as well as low oxygen conditions. The expression of *ADH1* genes, which is typically regulated by stress-related transcription factors such as ERF71 and ERF73 (Tan and Zwiazek, 2019), was up-regulated. The *LBD41* gene has been reported to be associated with hypoxia responses and linked to the ethylene pathway, such as under oil stress in Arabidopsis (Guedes et al., 2018). *HRA1* is usually induced by anaerobic conditions, leading to alcohol fermentation in plants (Abdeeva et al., 2018). The expression of *PDC1* is strongly up-regulated during flooding in rice (Hess et al., 2011; Zhang et al., 2016). Both cysteine dioxygenase (CDO) and cysteamine dioxygenase (ADO, 2-aminoethanethiol dioxygenase) are essential for taurine biosynthesis, and taurine acts as a biological O_2_ sensor. In plants, aspartate is oxidized by aspartate oxidase (AO) to produce iminoaspartate, ultimately leading to the synthesis of NAD^+^, which plays a critical role in maintaining metabolic balance (Saito et al., 2022). AO is also required for RBOHD-dependent stomatal closure, which limits gas exchange between plants and the atmosphere (Macho et al., 2012). Here, the up-regulation of genes *LBD41, HRA1, PDC1, ADO*, and *AO* implies that BCP limited gas exchange between the plant and the atmosphere, resulting in low oxygen levels inside the cells.

Lipoxygenase (LOX) is responsible for catalyzing the peroxidation of polyunsaturated fatty acids (Suzuki et al., 2015). In Petunia hybrida, PhCS plays a crucial role in the synthesis of chlorophylls, carotenoids, and anthocyanins (Masson et al., 2019). The F-BOX STRESS INDUCED (FBS) proteins have been associated with environmental stress networks, and WD40 repeat-like proteins are known targets of SCFFFBSI complexes (Sepulveda-Garcia et al., 2021). The up-regulation of *LOX, CS*, and *LOX3* genes suggested that BCP induced stress and damaged the metabolic pathway. These results were consistent with the studies of Wang, who have demonstrated that herbicides can regulate *LOX3, ADH1*, and *ERF* (Wang et al., 2021).

In plants, MYBs have various roles, such as regulating secondary cell-wall formation and participating in cell communication. *At5g28237* (*MYB95*) encodes tryptophan synthase (Xiao et al., 2021), which is responsible for synthesizing tryptophan, a precursor for IAA. Environmental stresses often cause an increase in H_2_O_2_ levels, leading to damage to plant cells, Glutathione S-transferase (GST) utilizes glutathione as a substrate to remove H_2_O_2_ (Horváth et al., 2020). In this study, the down-regulation of *At5g28237* indicated a decrease in IAA synthesis. Furthermore, the down-regulation of GST suggested that the application of BCP reduced the plant’s ability to withstand adverse environments.

### 4.3 Eugenol treatment inhibited cell division and decreased the protective effect against osmotic stress and oxidative stress

Eight hub genes were identified under treatment with eugenol, most of which were involved in cell division. Specifically, *CYCA, CYCB*, and *CYCD* are known to be involved in controlling the S-phase, G2-to-M-phase, and G1 phase in the cell cycle, respectively (Dante et al., 2014; Niu et al., 2015). *CDC20.1* is necessary for the separation of sister chromosomes. *TPX2* mediates the assembly and nucleation of microtubules, as well as mitotic spindle assembly in cell division (Tomastikova et al., 2020). Additionally, it is estimated that *NAC062* plays a role in regulating the cell cycle, relaying ER stress signaling from the plasma membrane to the nucleus, and regulating UPR downstream gene expression (Yang et al., 2014). Here, the upregulation of *CYCB1;3, NAC062*, and *TPX2* under eugenol treatment indicated that eugenol inhibited cell division and induced ER stress.

ACCase is known to play a pivotal role in fatty acid metabolism (Wang et al., 2018). Transcript changes of *ACCase* showed that eugenol affected fatty acid metabolism. In Arabidopsis, the enzyme HPPD (also known as PDS1) catalyzes the oxidative decarboxylation and rearrangement of p-hydroxyphenylpyruvate (HPP) to homogentisate (HGA), which is a precursor of tocopherols and plastoquinone, crucial co-factors in photosynthesis. Ahrens et al. (Ahrens, et al., 2013) stated that a mutant of *HPPD* displays albino and dwarf phenotypes with chlorophyll degradation. Downregulation of *PDS1* through eugenol exposure lead to bleaching of Arabidopsis, indicating that the synthesis of vital pigments and co-factors required for plant growth and protection against oxidative stress may be impacted by eugenol.

Exogenous trehalose regulates antioxidant production and osmotic balance to improve cold stress tolerance in rapeseed (Raza et al., 2022). Trehalose biosynthesis involves the conversion of Glc-6-P and UDP-Glc to trehalose-6-P by TPS. The *AtTPS8* gene is induced in leaves when sugar starvation occurs during extended night periods (Harthill et al., 2006). Eugenol treatment resulted in the down-regulation of *TPS8* genes, leading to dysfunction of sugar signaling at night or reduction in protection from osmotic stress.

### 4.4 The ability of these BCP and eugenol to target multiple genes through allelopathy allowed them to overcome tolerance to chemical herbicides and could serve as excellent candidates for natural biological herbicides

Natural herbicides derived from the allelopathy of compounds can provide effective alternatives to chemical herbicides for sustainable agricultural practices (Ahuja et al., 2015). In this study, we discovered that BCP and eugenol inhibited the growth of *A. thaliana* seedlings by affecting the expression of various genes involved in different metabolic pathways. The ability of these natural components to target multiple genes through allelopathy made them capable of overcoming tolerance to chemical herbicides. They induced oxidative stress pathways, disrupted the structure of the cell wall, and affected the plant’s endogenous hormone balance, thereby affecting its morphological phenotype. In terms of gene expression and regulation, BCP treatment primarily resulted in hypoxia stress, while eugenol caused cell division failure. In conclusion, BCP and eugenol have phytotoxic potential, making them excellent candidates for natural biological herbicides.

## 5 Conclusions

This study firstly provided molecular mechanism of inhibitory action of BCP and eugenol on plants. BCP and eugenol disrupted the endogenous hormone balance of plants, resulting in dysregulation of gene expression and regulation during growth and development. Among the hub genes responsive to BCP, several genes were up-regulated, including *HRA1*, *ADH1*, *ADO*, *ERF71* and *ERF73*, *PDC1*, and *LBD41*, all of which are related to hypoxia. Conversely, the down-regulated gene *GSTU5* is involved in detoxification, a crucial process for plant survival. Therefore, BCP causes excessive accumulation of ethylene and low oxygen conditions, disrupting metabolic pathways and reducing the plant’s ability to withstand adverse environments. It also impairs the plant’s detoxification abilities, leading to detrimental effects on plants in the long run. Regarding eugenol, four genes associated with cell division, *CDC20.1*, *Cyclin B1-3*, *TPX2*, and *AT5G55520*, were found to be down-regulated. Eugenol also down-regulated genes of ACCase and PDS1, involved in fatty acid metabolism and photosynthesis, respectively. These findings suggest that eugenol inhibits cell division and decreases the plant’s ability to survive. The continuous use of chemical herbicides has led to weed resistance and poses serious environmental threats. The application of BCP and eugenol could be an environmentally friendly and effective strategy for controlling resistant weeds.

## Data availability statement

The original contributions presented in the study are included in the article/**Supplementary Material**. Further inquiries can be directed to the corresponding author.

## Author contributions

YG: Conceptualization, Data curation, Funding acquisition, Methodology, Project administration, Supervision, Visualization, Writing – original draft, Writing – review & editing, Proofreading. CL: Writing – original draft, Writing – review & editing, Methodology, Software. YZ: Writing – original draft, Writing – review & editing, Methodology. SZ: Formal analysis, Software, Methodology. PC: Formal analysis, Software, Methodology. XW: Writing – review & editing, Methodology. TZ: Methodology, Writing – original draft, Writing – review & editing, Formal analysis, Proofreading.
