# Supplementary material for: Characterization key genes of Arabidopsis seedlings in response to β-caryophyllene, eugenol using combined transcriptome and WGCN analysis
Source: Front Plant Sci. 2024 Jan 4;14:1295779. doi: 10.3389/fpls.2023.1295779 (PMC10794411; doi:10.3389/fpls.2023.1295779)
Supplement: Supplementary file 1 [file DataSheet_1.docx]

**Supplemental Table S1. Primer sequence table of real-time fluorescence quantitative PCR**

| Gene name | Forward primer sequence (5’-3’) | Revers primer sequence （5’-3’） |
| --- | --- | --- |
| At-actin  AT1G72910 | TCCCAGTGTTGTTGGTAGGC  CACACATGAAGGAACTAAACCG | ATGGCGACATACATAGCGGG  CCTGAGAAATCGTTTTCACGTT |
| AT4G27280 | AGCTTTTGATGGACAGAGAGAA | AAAACTCCATCTGATTCAACGC |
| AT5G61600 | CAACACCTTCTCACAGACTTTG | TGTCTTTGTTGAAACCGTTCTC |
| AT1G18970 | CTGACTTCTTCTACTCCGGTTT | GCTAGCTCAACGTTATTCATCG |
| AT4G31970 | ACAGCTTCTTTACCGAACCTTA | GTTAGAAAGTAGCTCGAGGGTT |
| AT1G28680 | GTTTGGGACAGAGAACGTTTAC | AGTCTCTTTTGACGTCTCCAAT |
| AT3G26740 | CAACCAAAGCTTCAAGACTCTC | TTCAGCTGGAAAAACAGAAAGG |
| AT5G63530 | ACTGTGAAGGATGTGCTAGAAA | CCCCGTTTTACAATCAGTCATC |
| AT5G63190 | CAGAATCTTGCAAAGGGTTTCA | GCATCTACAAGCTTCGTAAGTG |
| AT5G16970 | GCCTATGCTGGGTTTTATGAAG | ATCATCTTAGCAAGTTGTCCCA |
| AT5G63790 | CTGATAAACCGATCGGAAAACC | CTCGTGCATAATCCAATTCGTT |
| AT2G08340 | CCCGGTTCAAAAACCCAAACC | CTTTTGGGAATGTGGGCGAG |
| AT2G29490 | CATCGATGACCAGATCCTAACA | CATGTCCAAGAATCCGATTGTC |
| AT3G28210 | CCTGTAAAACGTTGCAAAGAGA | CGGTGTTTCAAACAGAACTTGA |
| AT4G13180 | CAAGAGGGTCTTTCTTGTGTTG | GATGCTGCGTAAACTCCATAAC |
| AT3G53260 | TTGCTTAAGGTTGTTGATCGTG | GATCGAAGTCACTGCATTCTTC |
| AT5G40000 | AAGTCAGAGACGAGTACAAAGG | CCAAAAACCCATTGCGTACTTA |

**Supplemental Table S2. List of the Hub genes in different modules under BCP or eugenol treatment**

| Module | Gene ID | Gene name | GS | MM | Descrition | Gene expression |
| --- | --- | --- | --- | --- | --- | --- |
| salmon-  BCP | *AT3G25050* | *XTH3* | 0.7227989 | 0.8185088 | xyloglucan endotransglycosylase/hydrolase3 | up |
| salmon-  BCP | *AT3G06880* | *AT3G06880* | 0.825431 | 0.902529 | Transducin/WD40 repeat-like superfamily protein | down |
| salmon-  BCP | *AT2G29450* | *GSTU5* | 0.764293 | 0.894421 | glutathione S-transferase tau 5 | down |
| salmon-  BCP | *AT1G17420* | *LOX3* | 0.751216 | 0.805503 | lipoxygenase 3 | down |
| salmon-  BCP | *AT1G74430* | *MYB95* | 0.713505 | 0.911604 | myb domain protein 95 | down |
| salmon-  BCP | *AT5G28237* | *AT5G28237* | 0.711406 | 0.943692 | Pyridoxal-5'-phosphate-dependent enzyme family protein | down |
| brown-  BCP | *AT2G34390* | *NIP2.1* | 0.972513 | 0.893197 | NOD26-like intrinsic protein 2.1 | up |
| brown-  BCP | *AT3G10040* | *HRA1* | 0.940113 | 0.91641 | hypoxia response atteuator1 | up |
| brown-  BCP | *AT1G77120* | *ADH1* | 0.935953 | 0.862373 | alcohol dehydrogenase 1 | up |
| brown-  BCP | *AT5G15120* | *DUF1637* | 0.901078 | 0.958007 | 2-aminoethanethiol dioxygenase, putative | up |
| brown-  BCP | *AT1G48850* | *EMB1144* | 0.895275 | 0.975694 | chorismate synthase, | up |
| brown-  BCP | *AT3G02550* | *LBD41* | 0.890961 | 0.928044 | LOB domain-containing protein 41 | up |
| brown-  BCP | *AT4G33070* | *PDC1* | 0.868936 | 0.875173 | pyruvate decarboxylase 1 | up |
| brown-  BCP | *AT5G14760* | *AO* | 0.854319 | 0.938729 | L-aspartate oxidase | up |
| brown-  BCP | *AT1G72360* | *ERF073* | 0.802264 | 0.920182 | ethylene response fator 73 | up |
| brown-  BCP | *AT2G47520* | *ERF071* | 0.747439 | 0.868578 | ethylene response fator 71 | up |
| darkred-  Eugenol | *AT3G11520* | *CYCB1-3* | -0.82827 | 0.833752 | cyclin B1.3 | down |
| darkred-  Eugenol | *At1G03780* | *TPX2* | -0.75677 | 0.796924 | targeting protein for XKLP2 | down |
| darkred-  Eugenol | *AT5G55520* | *AT5G55520* | -0.75354 | 0.893898 | kinesin-like protein | down |
| sienna3-  Eugenol | *AT1G36180* | *ACC2* | 0.872012 | 0.857785 | acetyl-CoA carboxylase 2 | up |
| sienna3-  Eugenol | *AT4G13395* | *RTFL12* | 0.80193 | 0.915701 | rotunaifolia like 12 | up |
| sienna3-  Eugenol | *AT3G49530* | *NAC062* | 0.791448 | 0.90262 | NAC domain containing protein 62 | up |
| sienna3-  Eugenol | *AT1G06570* | *PDS1* | 0.761972 | 0.968337 | 4-hydroxyphenylpyruvate dioxygenase | up |
| sienna3-  Eugenol | *AT1G70290* | *TPS8* | -0.76122 | 0.88371 | trehalose-6-phosphatase synthase S8 | down |

##
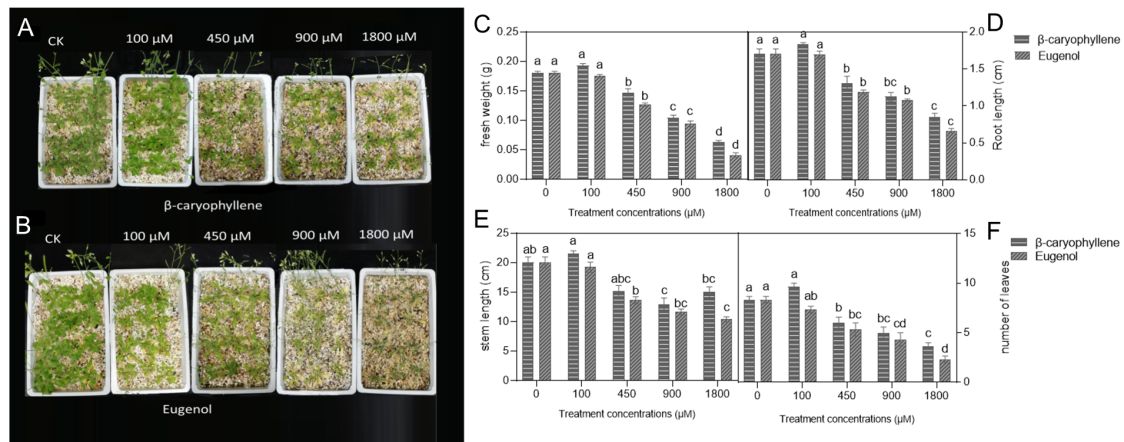


Supplementary Fig. 1. A. thaliana seedlings in response to BCP (A) or eugenol (B); The fresh weight (C), root length (D), stem length (E) and number of leaves (F) in A. thaliana seedlings in response to BCP or eugenol.


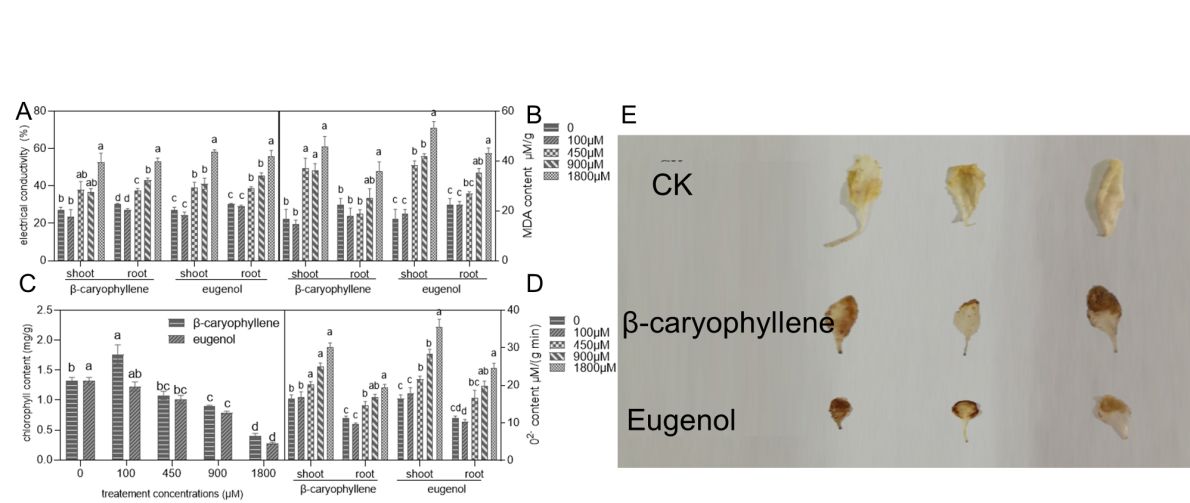


Supplementary Fig. 2. The electrical conductivity (A), MDA (B) contents, chlorophyll (C), O^-^_2_(D) and H_2_O_2_ (E) of shoots and roots in A. thaliana seedlings after treatment with BCP or eugenol.


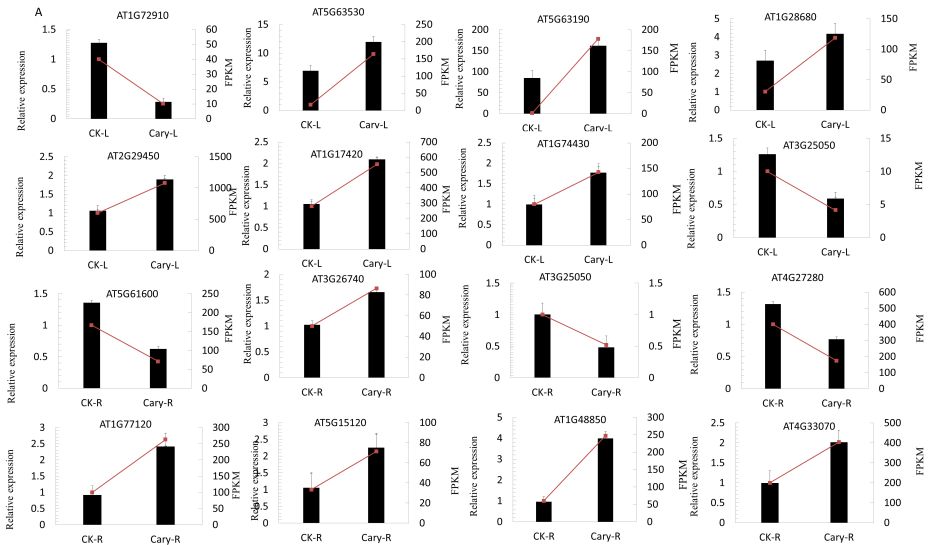


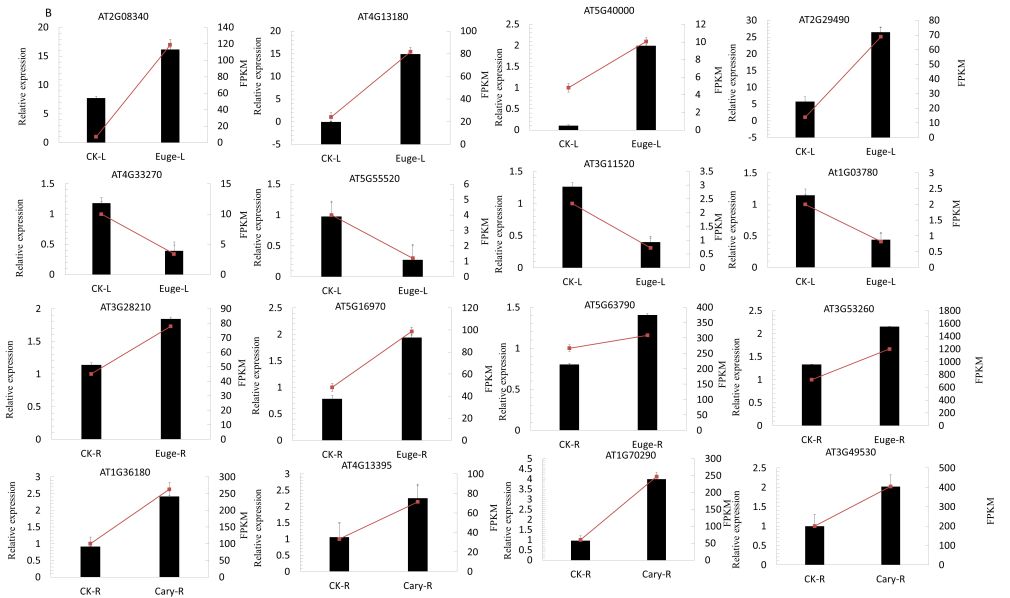


Supplementary Fig. 3. qRT-PCR analysis of DEGs in Arabidopsis thaliana seedlings after BCP treatment A, expression levels revealed of shoot (A) and root (B) in Arabidopsis thaliana seedlings using qRT-PCR and RNA-seq of randomly selected DEGs. The Y-axis on the left shows therelative gene expression levels analyzed using qRT-PCR (red lines) with 3 replicates, while the Y-axis on the right shows thecorresponding expression data for RNA-seq.
